# The association between smartphone addiction and creativity in Chinese college students: the chain mediating effects of depression and executive function

**DOI:** 10.1186/s12888-025-07378-y

**Published:** 2025-09-30

**Authors:** Wenfu Li, Jinmei Liu, Xia Liu, Yao Ge, Yan Wang, Aoxue Zhang

**Affiliations:** 1 School of Rehabilitation Medicine, Jining Medical University, Jining, Shandong China; 2 School of Mental Health, Jining Medical University, Jining, Shandong China

**Keywords:** Creativity, Smartphone addiction, Depression, Executive function

## Abstract

**Background:**

Smartphone addiction has emerged as a widespread public health concern, especially among college students. This study examines the association between smartphone addiction and creativity among Chinese college students, as well as the underlying psychological mechanisms involved. A serial mediation model framework was conducted to explore the effect of smartphone addiction on creativity, taking into consideration the mediating roles of depression and executive function.

**Methods:**

In this study, a sample of 691 Chinese college students was surveyed using the Mobile Phone Addiction Index, the Centre for Epidemiologic Studies - Depression Scale, the Behavior Rating Inventory of Executive Function - Adult Version, and the University Students’ Creativity Scale to examine the impact mechanism of smartphone addiction on creativity. Correlation and mediation model analyses were performed respectively using SPSS 22.0 and PROCESS 3.1.

**Results:**

Correlation analysis showed that smartphone addiction was positively correlated with depression (*r *= 0.298, *P *< 0.001) and executive function impairment (*r *= 0.677, *P *< 0.001), while it was negatively associated with creativity (*r *= -0.209, *P *< 0.001). Additionally, depression was positively correlated with executive function impairment (*r *= 0.376, *P *< 0.001). Furthermore, creativity was negatively associated with depression (*r *=-0.143, *P *< 0.001) and executive function impairment (*r *= -0.291, *P *< 0.001). The mediation analysis indicated that smartphone addiction directly negatively predicted creativity, and also indirectly predicted creativity through the serial mediating role of depression and executive function impairment.

**Conclusion:**

Smartphone addiction not only directly affected creativity, but also indirectly influenced it through both the independent mediating role of executive function, as well as their serial mediation pathway.

## Instruction

Smartphones have deeply penetrated every aspect of our lives and transformed the way we live. According to the report from the China Internet Network Information Center [1], there were approximately 1.1 billion smartphone users in China by the end of 2024. While smartphones have revolutionized communication, entertainment, and productivity, their overuse can lead to smartphone addiction [2], which negatively impacts health outcomes [3], social interactions [4], and even well-being [5]. Other studies have also found that smartphone addiction increases the risk of sleep problems, anxiety, depression, and poor academic performance [6, 7]. Consequently, smartphone addiction has garnered considerable attention from governments, societies, and scholars.

Creativity, which plays a pivotal role in personal growth and social progress, can be defined as the ability to generate ideas or solutions that are both novel and useful [8, 9]. Although there are plenty of studies on the association between smartphone use and creativity, the findings are mixed [10]. China, as the country with the largest number of smartphone users in the world (with over 1.1 billion smartphone users), can provide researchers with important data resources for analyzing the relationship between smartphone addiction and creativity among adolescents. However, based on a comprehensive literature review, no empirical study has yet examined this topic. Therefore, the present study aimed to investigate the association between smartphone addiction and creativity among Chinese college students through a cross-sectional design, and to explore the mediating role of depressive symptoms and executive function.

### The relationship between smartphone addiction and creativity

Smartphone addiction is defined as uncontrolled smartphone use causing detrimental effects on physical health, mental health, and social functioning [11]. Numerous studies have examined the association between smartphone use and creativity [12, 13]. For example, as Wilmer et al. [14] pointed out that public opinion holds that smartphones distract people’s attention and thus hinder creativity. Aru and Rozgonjuk [15] also contended that disruptive habitual smartphone use could have an adverse effect on the sustained cognitive effort essential for creative achievement. Furthermore, a recent survey research conducted three correlational studies to explore the correlation between smartphone use and creativity, and found that problematic smartphone use negatively correlated with creativity as measured by divergent thinking and creative achievement [12]. Previous study has indicated that excessive smartphone use may lead to smartphone addiction [16]. Therefore, the evidence suggests that smartphone addiction might be negatively correlated to creativity, as excessive smartphone use may interfere with creativity.

The “brain drain” hypothesis points out that the mere presence of one’s smartphone can occupy limited-capacity cognitive resources, thereby reducing available resources for other tasks and impairing overall cognitive performance such as problem solving and creativity [17]. Indeed, recent empirical studies have demonstrated that smartphone addiction is significantly associated with reduced creativity. For example, Müller and Montag [10] examined the association between smartphone use disorder and creative self-efficacy as measured by the three-item Creative Self-Efficacy Scale [18], and found that smartphone use disorder was negatively associated with creative self-efficacy. Furthermore, Guo et al. [19] found that smartphone addiction was negatively related with creativity, as measured by the Creativity Self-evaluation Scale, in a sample of 2,900 Chinese college students. Therefore, there is reasonable evidence to suggest that smartphone addiction negatively predicts creativity. Nevertheless, the mediating processes driving this association have yet to be elucidated.

### The mediating role of depression

Depression, as a widespread psychological issue, is characterized by the prolonged periods of sadness or a persistent loss of interest and pleasure in activities [20]. A concerning upward trend in depressive symptoms among college students has emerged globally, with current prevalence rates even higher than those observed in the general population [21]. A recent study reported that subthreshold depression has a prevalence rate of approximately 40% among Chinese university students [22]. Other studies also reported that the depressive symptoms prevalence rate of 29.7% among Chinese freshmen [23], and the prevalence of depressive symptoms among Chinese university students during the COVID-19 pandemic was 26.0% [24]. The high prevalence of depressive symptoms among Chinese university students highlights the critical need to investigate their mediating role in linking early-life adversity to subsequent psychological and behavioral impairments.

Numerous studies suggested that depression is influenced by multiple factors, among which problematic smartphone use has been identified as a significant potential risk factor [25, 26]. Furthermore, a significant positive correlation between smartphone addiction and depression has been found among 2,469 secondary school students [27]. Additionally, a meta-analysis of 27 studies involving 120,895 participants revealed a significant positive association between smartphone addiction and depression [28]. Moreover, existing research also demonstrated that depressive symptoms may contribute to the development of smartphone addiction [29]. These findings may suggest a bidirectional relationship between smartphone addiction and depressive symptoms [30, 31]. Therefore, these studies consistently demonstrate a positive association between smartphone addiction and depressive symptoms. Given the high prevalence of both smartphone addiction and depressive symptoms, investigating their psychological and behavioral impacts holds significant public health importance.

Depression often involves low energy, lack of motivation, sleep disturbances, and intense feelings of sadness, inadequacy, and despair [32]. Numerous studies have supported a negative relationship between depression and creativity [33]. Delpech et al. (2017) found a negative correlation between depression and creativity in the cancer patient population. Soeiro-de-Souza et al. [34] investigated the relationship between creativity and different symptomatic phases of bipolar disorder (BD), including manic, mixed, and depressive states, and found that patients in manic or mixed states demonstrated significantly higher creativity scores compared to those experiencing depressive episodes. A systematic review encompassing 12 studies also revealed that creative performance is significantly impaired during depressive phases of BD [35]. Based on these studies, we can hypothesize that depression may mediate the association between smartphone addiction and creativity.

### The mediating role of executive function

Executive function refers to a set of domain-general cognitive control processes that modulate both mental operations and behavioral responses [36]. The Diamond’s model [37] indicates that executive function consists of three core components: inhibitory control (the capacity to deliberately inhibit task-inappropriate behavioral responses), cognitive flexibility (the capacity to flexibly switch between distinct cognitive sets or tasks), and working memory updating (the capacity to update and regulate the contents of working memory). Previous studies have examined the association between smartphone use/addiction and executive functions, which are widely recognized as critical cognitive components for creativity [38–40]. Growing clinical evidence also indicates a strong association between behavioral addictions and measurable impairments in executive functions [41, 42]. Hartanto and Yang [39]found that both smartphone separation and smartphone addiction have adverse effects on executive function. León Méndez et al. [38] also indicated that both internet addiction and smartphone addiction were related to the impaired executive function using functional magnetic resonance imaging. Therefore, we can hypothesize that smartphone addiction can negatively predict executive function performance in college students.

Additionally, there is increasing evidence that executive functions play a role in creativity. It has been indicated that executive function (cognitive flexibility or cognitive switching) demonstrates a stronger predictive effect on creativity than fluid and crystallized intelligence [43].Zabelina et al. [44] found that working memory updating positively predicted the fluency score of divergent thinking tasks, and response inhibition positively predicted the number of real-world artistic creative achievements. Other studies have identified associations between executive functions and creativity across diverse populations [43, 45, 46]. The Dual Pathway Creativity Model (DPCM) proposes that creativity is shaped by the interplay between cognitive flexibility and cognitive persistence [47]. Various studies have found that both cognitive flexibility and cognitive persistence are positively correlated with creativity [48–50]. More direct evidence is provided by studies showing that executive function training promotes creativity [46, 51]. Furthermore, studies have shown that executive function mediates the relationship between certain variables (e.g., socioeconomic status and bilingualism) and creativity [45, 52]. Based on these research findings, we can assume that executive function possibly mediates the relationship between smartphone addiction and creativity.

### The serial mediating role of depression and executive function

Depression is not only the most prevalent mental health problem [53], but it also significantly impacts individuals’ cognitive abilities [54]. Executive functioning is one of the cognitive areas most impaired by depression [55]. Clinical studies have found that patients with depression exhibit impairments in executive function [56]. A cross-sectional study of children aged 7–12 years revealed a significant inverse association between depressive symptoms and executive function performance [57]. Rajtar-Zembaty et al. [58] also revealed significant impairments in all executive function domains among individuals with late-life depression. A longitudinal study further demonstrated that elevated depressive symptoms predict future declines in executive function in adults [59]. Furthermore, the finding that depression leads to impaired executive function is further supported by neuroimaging evidence [60]. Therefore, we have reason to believe that depression and executive function could sequentially mediate the association between smartphone addiction and creativity.

### The present study

**Fig. 1:**
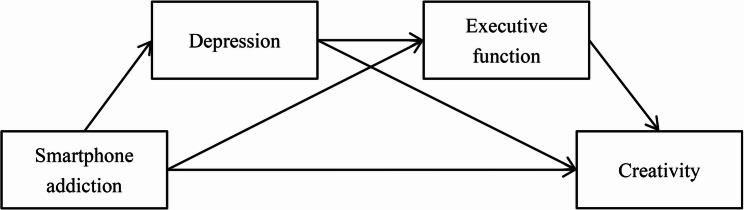
The hypothesized model of the chain mediating role of depression and executive function in the relationship between smartphone addiction and creativity

In conclusion, the current study has two aims. First, we aimed to investigate the association between smartphone addiction and creativity. Based on previous findings, we hypothesized that smartphone addiction would positively predict creativity. Second, we aimed to examine the possible mechanism underlying the association between smartphone addiction and creativity. Based on the relationship among smartphone addiction, depression, executive function, and creativity, we proposed a serial mediation model where depression and executive function act as mediators sequentially in the relationship between smartphone addiction and creativity. The conceptual model hypothesized that a more severe smartphone addiction would be related to more intense depressive symptoms, which would then be associated with greater impairment in executive function; the greater impairment in executive function would, in turn, be related to a poorer creative performance. Specifically, smartphone addiction may correlate with creativity through both a direct pathway and three indirect mediating pathways: smartphone addiction → depression → creativity, smartphone addiction → executive function → creativity, and smartphone addiction → depression → executive function → creativity. The hypothesized variable relationships are graphically represented in Fig. 1, and the hypothesized model was tested using an online survey administered to a large sample of college students.

## Methods

### Subjects

In the present study, the convenience sampling method was used to recruit college students from a university in Jining, a city in Shandong province, China. The electronic questionnaires were distributed to subjects using Sojump (https://www.wjx.cn/) — a reliable and widely used Chinese online survey platform. Before filling out the questionnaires, all participants provided informed consent. A total of 729 questionnaires were distributed, and 38 ones were excluded Due to excessively short response times and missing answers. Totally 691 valid questionnaires remained, and the effective rate was 94.79%. The participants included 322 (46.47%) males and 369 (53.53%) females; 339 (49.07%) from rural areas and 352 (50.93%) from urban areas; 244 (35.29%) only children and 447 (64.71%) non-only children.

### Measures

#### Demographic information

The Demographic Information Form (DIF) was used to collect demographic information from the participants. The information includes: gender, age, residence, and sibling status (only child vs. with siblings).

#### Smartphone addiction

The Mobile Phone Addiction Index (MPAI), originally developed by Leung [61] and translated into Chinese by Huang et al. [62], was used to measure the level of smartphone addiction among college students, which was. This scale consists of 17 items, with typical examples such as “You have attempted to spend less time on your mobile phone but are unable to”. This scale adopts a 5-point-Likert scale with 1 = not at all and 5 = always. This scale has good reliability and validity (Huang et al., 2014). In this study, the coefficient α was 0.879.

#### Depression

The Centre for Epidemiologic Studies-Depression Scale(CES-D)was used to measure depressive symptoms, which was originally developed by Radloff [63] and translated into the Chinese version by Chen et al. [64]. This scale includes 20 items, which include items such as “I thought my life had been a failure”. Each item was answered on a 4-point Likert scale with 0= rarely or none of the time and 3 = most or all of the time. There is extensive evidence for the high internal consistency and measurement invariance in college students and clinical patients [65]. In this study, the coefficient α was 0.835.

#### Executive function

The Behavior Rating Inventory of Executive Function - Adult Version (BRIEF-A) was employed for the assessment of executive function, which was designed by Roth et al. [66] and adapted into the Chinese version by Du et al. [67]. This scale consists of 75 items, with typical examples such as “I have trouble organizing activities”. The answers of this scale adopt a 3-point Likert scale with 1= never and 3 = always. Higher scores represent lower executive function. It was shown that this measure has excellent internal consistency [66, 68]. In this study, the coefficient α was 0.976.

#### Creativity

The University Students’ Creativity Scale (USCS) was used to assess creativity, which was developed and validated by He et al. [69] with a sample of Chinese college students. This scale includes 16 items, which include items such as “I like to ask questions that others haven’t thought of”. All of the items were answered on a 5-point Likert scale with 1= strongly disagree and 5 = totally agree. Higher scores indicate a stronger creative tendency. In this study, the coefficient α was 0.785.

#### Procedure

In this study, we used the smartphone addiction, depression, executive function, and creativity scales to collect data through an online survey. All subjects agreed to the informed consent form before they started to do this online survey. After participants completed all the items, the test data were auto-generated. The procedure was approved by the research ethics committee of Jining Medical University.

### Statistical analysis

SPSS 22.0 was used to conduct the descriptive analysis, correlation analysis, common method bias test, and reliability analysis. SPSS PROCESS 3.3 [70] Macro was used for the chaining mediation model analysis.

## Results

### Common method bias test

Self-administered surveys may lead to the common method bias which can influence the reliability and validity of the results [71]. In this study, Harman’s single factor analysis was used to test the common method bias and all the items of MPAI, CES-D, BRIEF-A, and USCS were entered into the exploratory factor analysis. The results showed that there were 26 components with initial eigenvalues greater than 1. The first component accounted for 25.04% of the variance, which was less than the critical value of 40%, indicating that there was no significant common method bias [72].

### Descriptive statistics and correlation

Descriptive data (means, standard deviations, and correlation coefficients) of all variables are listed in Table 1. We used Pearson correlation analysis to examine the bivariate correlations of all variables. Results showed significant positive associations among smartphone addiction, depression, and executive function. Meanwhile, creativity was significantly negatively associated with smartphone addiction, depression, and executive function.

**Table 1:** The results of descriptive statistics and correlations of all variables

Variable	M	SD	1	2	3
1.Smartphone Addiction	45.75	12.22			
2.Depression	18.32	10.61	0.289***		
3.Executive Function	124.56	38.69	0.677***	0.376***	
4.Creativity	55.87	8.11	−0.209***	−0.143***	−0.291***

*N* = 691

***P<0.001

### The chain mediating effects analysis

The correlation analysis results showed that smartphone addiction, depression, executive function and creativity significantly correlated with each other, which meets the statistical requirements for further chain mediating effect analysis of smartphone addiction and creativity [73]. After controlling for Gender, age, residence, and only-child status, the Model 6 in PROCESS 3.3 [70] was used to analyze the chain mediating role of depression and executive function in the association between smartphone addiction and creativity. All variables in the model had been standardized.

**Fig. 2:**
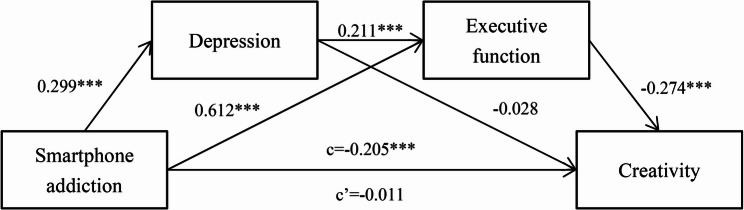
The chain mediating effect of depression and executive function in the association between smartphone addiction and creativity. Note: The data in the figure represent standardized path coefficients; ***, *P*<0.001; c, the direct effect of smartphone addiction on creativity; c’, the indirect effect of smartphone addiction on creativity.

The regression analysis results of the association between smartphone addiction and creativity are listed in Table 2; Fig. 2. The results show that smartphone addiction has a significant negative predictive effect on creativity (*β* = −0.205, *P <* 0.001). When depression and executive function are included in the regression model, smartphone addiction significantly predicts depression (*β* = 0.299, *P* < 0.001) and executive function (*β* = 0.612, *P <* 0.001). Depression significantly predicts executive function (*β* = 0.211, *P <* 0.001) nor creativity (*β* = −0.028, *P* > 0.05). In addition, executive function is a significant negative predictor of creativity (*β* = −0.274, *P <* 0.001). At this point, the direct effect value of smartphone addiction on creativity is no longer statistically significant (*β* = −0.011, *P >* 0.05). These results indicate that the single mediating effect of executive function and the chain mediating effect of depression→executive function are statistically significant in the influence of smartphone addiction on creativity.

**Table 2:** Regression analysis of the relationship between smartphone addiction and creativity

Predictor variable	Model 1 (Creativity)		Model 2 (Depression)		Model 3 (Executive function)		Model 4 (Creativity)	
	β	t	β	t	β	t	β	t
Gender	0.058	1.549	−0.167	−4.631***	0.068	2.482*	0.062	1.673
Age	−0.058	−1.561	−0.060	−1.669	0.007	0.249	−0.061	−1.684
Residence	−0.084	−2.174	0.014	0.378	−0.020	−0.708	−0.088	−2.335*
Only-child	0.001	0.016	−0.020	−0.531	0.042	1.481	0.010	0.273
Smartphone addiction	−0.205	−5.500***	0.299	8.302***	0.612	21.484***	−0.011	−0.231
Depression					0.211	7.333***	−0.028	−0.695
Executive function							−0.274	−5.346***
Creativity								
* R* ^2^	0.057		0.116		0.500		0.101	
* F*	8.318***		17.997***		114.056		11.002***	

The mediating roles of depression and executive function in the link between smartphone addiction and creativity are summarized in Table 3. The results show that depression and executive function play a significant mediating role between smartphone addiction and creativity. The total effect value of smartphone addiction on creativity is −0.2049, the direct effect value of smartphone addiction on creativity is −0.0114, and the total indirect effect value is −0.1935. The ratio of the total indirect effect to the total effect is 94.44%. The mediating effect includes three indirect effects. The indirect effect value of path 1 (smartphone addiction→depression→creativity) is −0.0083, the indirect effect value of path 2 (smartphone addiction→executive function→creativity) is −0.1678, and the indirect effect value of path 3 (smartphone addiction→depression→executive function→creativity) is −0.0174. The ratios of the three paths to the total effect are 4.05%, 81.90%, and 8.49% for indirect effects 1, 2, and 3 respectively. Paths 2 and path 3 are statistically significant because the 95% confidence interval does not contain the zero value, and the path 1 is not statistically significant because the 95% confidence interval contains zero.

**Table 3:** Depression and executive function in the chain mediating effect model

	Effect	Boot SE	Boot LLCI	Boot ULCI	Effect Size
Total effect	−0.2049	0.0372	−0.2780	−0.1317	100%
Direct effect	−0.0114	0.0494	−0.1085	0.0857	5.56%
Total indirect effect	−0.1935	0.0368	−0.2679	−0.1237	94.44%
indirect effect 1	−0.0083	0.0121	−0.0324	0.0160	4.05%
indirect effect 2	−0.1678	0.0328	−0.2356	−0.1064	81.90%
indirect effect 3	−0.0174	0.0047	−0.0277	−0.0093	8.49%

*N* = 691

*Boot SE* Boot standard error, *Boot LLCI* Boot lower Limit of the 95% confidence interval, *Boot ULCI* Boot upper Limit of the 95% confidence interval

Indirect effect 1: smartphone addiction→depression→creativity

Indirect effect 2: smartphone addiction→executive function→creativity

Indirect effect 3: smartphone addiction→depression→executive function→creativity

## Discussion

With the fast development of digital technology in China [74], this study was conducted to investigate the association between smartphone addiction and creativity among Chinese college students. It further examined the serial mediating role of depression and executive function, which was hypothesized to mediate the relationship between smartphone addiction and creativity. The results showed that smartphone addiction was negatively associated with creativity. Executive function partially mediated the relationship between smartphone addiction and creativity. Furthermore, depression and executive function played a serial mediating role in the association between smartphone addiction and creativity. The current findings are consistent with previous studies demonstrating significant associations among smartphone addiction, depressive symptoms, executive function, and creativity [15, 43, 58, 75, 76]. The findings advance our comprehension of the psychological and cognitive mechanisms that underpin the association between smartphone addiction and creative cognition. The results could facilitate the design of more effective prevention and treatment strategies for college students’ smartphone addiction, fostering both psychological well-being and creative performance.

### Smartphone addiction correlated with creativity

The results found that smartphone addiction was negatively related to creativity, which supported our initial hypothesis and aligned with recent reports by Gao et al. [77]. Consistent with this, Guo et al. [19] reported that smartphone addiction negatively predicted creativity in 2,900 Chinese university students. Similarly, in a survey study including three correlation studies, Olson et al. [12] found both screen time and problematic smartphone use negatively correlated with divergent thinking, and smartphone use was weakly negatively associated with measures of divergent thinking or creative achievement. These findings indicate that both smartphone addiction and general smartphone use correlate not only with self-report creativity but also with objective behavioral assessments of creativity. Additionally, according to the attentional resource theory [78, 79], smartphone addiction makes people rely too much on their phones, spending excessive time on social media, games and other apps, which reduces their focus on real-life creative activities, weakens their curiosity for learning, and limits innovative thinking [19]. Empirical research also indicated that individuals with smartphone addiction exhibit impaired cognitive control [80], which hinders the generation of creative ideas [81]. Meanwhile, both social media and smartphone overuse have been demonstrated to trigger anxiety and cause ego depletion [25, 82], subsequently reducing creativity [83, 84]. Furthermore, the adverse impact of smartphone addiction on creativity has been validated by neuroimaging studies [85]. Therefore, smartphone addiction may impair creativity by depleting attentional resources, compromising cognitive control, inducing ego depletion, and even altering functional connectivity patterns in the brain.

Notably, while cross-cultural differences exist in smartphone usage patterns between Chinese and Western populations [86, 87], the negative association between smartphone addiction and creativity extends beyond Chinese college students. Existing research has validated this association among both western samples [12] and international students in China [77]. These findings indicated that there is cross-cultural consistency in the negative association between smartphone addiction and creativity. Therefore, this study provides further evidence that smartphone addiction leads to reduced creativity measured by self-reported questionnaires, enriching our understanding of the relationship between addictive behaviors and creative capacity.

### The mediating effect of depression

The present study identified significant correlations between smartphone addiction and depression, as well as between depression and creativity, but the mediating effect of depression in the relationship between smartphone addiction and creativity was not statistically significant. These results replicate prior research documenting both the positive association between smartphone addiction and depressive symptoms [27], and the inverse relationship between depressive symptoms and creative performance [33]. Extensive evidence confirms that smartphone addiction positively correlates with negative emotional states (e.g., anxiety, depression) [28, 88], which in turn have been shown to impair creative performance [84, 89]. Additionally, depression and other negative affective states are strongly associated with rumination [90], characterized by persistent and intrusive negative thinking [91]. This cognitive process depletes executive resources, consequently impairing creative potential [92]. The consistent pattern of findings lends support to the idea that college students with smartphone addiction may exhibit depressive symptoms, which in turn exert negative effects on creativity.

It should be noted that the present study did not identify a statistically significant mediating role of depression in the relationship between smartphone addiction and creativity. These findings are inconsistent with previous research [93], which confirmed the significant mediating role of depression in the association between smartphone addiction and creativity. This inconsistency might be attributed to methodological variations in depression and creativity assessments between the two studies. Cheng and Xie [93] used the depression subscale of the Depression, Anxiety, and Stress Scale (DASS) [94] to measure depressive symptoms and the Individual Innovation Scale (IIS) [95] to assess creativity. The DASS depression subscale measures depression primarily through low mood and feelings of worthlessness [94], whereas the CES-D assesses depressive symptoms across four domains: emotional distress, somatic complaints, interpersonal difficulties, and reduced positive affect (reverse-scored) [63]. The IIS is commonly used to assess creativity in the fields of organizational behavior and innovation management [96], whereas the USCS is specifically designed to measure creativity among college students [69]. Additionally, the inconsistent findings may also stem from the fact that the mediating role of depression between smartphone addiction and creativity could be moderated by other variables. For example, Cheng and Xie [93] found that the negative association between depression and creativity was significantly stronger under high rumination levels compared to low rumination conditions. Therefore, the mediating role of depression in the relationship between smartphone addiction and creativity may need to be further examined in future research.

### The mediating role of executive function

The results showed that executive function partially mediated the association between smartphone addiction and creativity. These findings are consistent with prior studies demonstrating that smartphone addiction/excessive use is linked with executive dysfunction [38–40], which in turn was linked to lower creativity [45, 52]. Numerous studies have demonstrated that both substance and non-substance addictions are associated with executive function deficits [39, 41, 97]. Zabelina et al. [44] further found that working memory updating predicted divergent thinking fluency, response inhibition correlated with artistic achievement, and artists outperformed non-artists in basic executive function and cognitive flexibility. Furthermore, neuroimaging research has found that smartphone addiction leads to impairments in executive function-related brain regions (e.g., DLPFC, frontal, and parietal lobes) in adolescents and young adults [38], and these brain regions have been found to be closely associated with creativity [98]. Our findings suggest that heightened smartphone addiction among college students is correlated with impaired executive function, which in turn is associated with diminished creative performance. The present study highlights the importance of intervening in executive dysfunction among individuals with smartphone addiction to mitigate adverse effects on creative performance.

Notably, research also indicated that digital games can foster creativity through their positive effects on executive function development and neuroplasticity [99]. For example, a study conducted by Jackson et al. [100] involving nearly 500 12-year-olds demonstrated a positive correlation between video game engagement and creative performance, particularly in picture drawing and story writing tasks. Another systematic review by Rahimi and Shute [101] revealed that while not all video games equally enhance creativity, those promoting flow states, player co-creation, and intrinsic motivation demonstrate the strongest creative potential. Furthermore, research has also demonstrated that game-based learning significantly enhances students’ creative capabilities relative to traditional non-game learning approaches [102]. Therefore, if smartphone use is regulated to avoid addiction, mobile platforms may serve as effective tools for creative interventions.

### The serial mediating role of depression and executive function

This study further found a significant pathway of smartphone addiction→depression→executive function→creativity. This model indicated that the chain association between depression and executive function mediated the relationship between smartphone addiction and creativity. A large number of studies have shown that the stronger smartphone addiction will lead to worse depressive symptoms among college students [26, 75], and there is a close positive association between depressive symptoms and the impaired executive function [103–105], as well as between good executive function and more creative thinking or behavior [37, 44]. The current study indicated that combined treatment of depression and executive function deficits in individuals with smartphone addiction was more effective for enhancing creativity than interventions focusing solely on addiction control.

#### Theoretical and practical implications

First, the current study established a sequential mediation pathway (smartphone addiction → depression → executive dysfunction → reduced creativity) that elucidates the underlying mechanisms through which smartphone addiction may impair creative capacities. This pathway extended existing theoretical frameworks by integrating affective (depression) and cognitive (executive dysfunction) mediators into a unified model. Our findings bridged previously distinct research domains by demonstrating the interconnectedness among behavioral addiction (smartphone abuse), mood disorders (depression), and cognitive function (executive function and creativity), highlighting the need for more integrated theoretical approaches in future research.

Second, college students are very valuable human resources, and they are the future of our society. Their creative thinking and innovative consciousness have been widely concerned by the governments around the world. Our findings suggest that dual interventions targeting both depressive symptoms and executive functioning in college students may preserve creative development more effectively than interventions focusing solely on smartphone addiction. Additionally, given the identified serial mediation involving depressive symptoms and executive dysfunction, educators should attach importance to depression screening among individuals with smartphone addiction and incorporate executive function training (e.g., working memory tasks, cognitive flexibility exercises) into treatment protocols. Moreover, both parents and educators should focus on adolescents’ smartphone usage habits to reduce the risk of smartphone addiction and facilitate the development of their creative potential.

#### Limitations and future directions

This study also has some limitations that need to be acknowledged. Firstly, it should be noted that the exclusive reliance on questionnaire data from a single university may constrain the generalizability of our findings. Future research could recruit participants from diverse universities, age groups, and cultural/national backgrounds to more systematically examine the relationship between smartphone addiction and creativity. Secondly, although the proposed serial mediation model between smartphone addiction and creativity was theoretically grounded in prior literature and empirical evidence, the cross-sectional questionnaire design precludes definitive causal inferences. Future longitudinal or experimental designs would be required to confirm the causal relationship between smartphone addiction and creativity. Thirdly, this study examined the relationship between smartphone addiction and creativity using self-report questionnaires, which may introduce response biases like social desirability bias [106] that could affect the conclusion. Future studies could employ multi-method assessments, such as third-party rating scales and behavioral tasks, to examine these relationships, and may further utilize neuroimaging techniques to validate these findings. Furthermore, this study only explored the mediating effects of depression and executive function impairment in the association between smartphone addiction and creativity. Future research could explore the mediating or moderating roles of additional variables (e.g., self-efficacy, self-esteem) in the relationship between smartphone addiction and creativity among college students, to provide a more comprehensive understanding.

## Conclusion

This study significantly advances our understanding of the sequential mediation pathway linking smartphone addiction, depression, executive dysfunction, and creativity among Chinese college students. Our findings confirmed that smartphone addiction is related to creativity through the serial mediating role of depression and executive dysfunction. This model suggests that governments and educational institutions should provide adequate support to college students. While it is challenging to alter students’ smartphone usage habits directly, we can intervene in the pathway between smartphone addiction and creativity by modifying these mediating factors to enhance creative performance. This dual-path approach may effectively mitigate the negative impact of smartphone addiction while fostering students’ creative development.

## Data Availability

Data will be made available on request.
